# The Management of a C1-Shaped Canal Configuration in a Mandibular Second Molar

**DOI:** 10.7759/cureus.66728

**Published:** 2024-08-12

**Authors:** Namrata P Jidewar, Manoj Chandak, Swayangprabha Sarangi, Paridhi Agrawal, Tejas Suryawanshi, Kapil M Naladkar, Palak Hirani

**Affiliations:** 1 Department of Conservative Dentistry and Endodontics, Sharad Pawar Dental College, Datta Meghe Institute of Higher Education and Research, Wardha, IND

**Keywords:** backfill, thermoplasticized, endodontics, root canal treatment, mandibular molar, c shaped canal

## Abstract

The mandibular second molar has one unique feature regarding internal anatomy: it tends to have a C-shaped configuration in many cases. In mandibular second molar teeth, it is a variance of racial predilection that is frequently observed. During negotiation, debridement, and obturation, the physician is frequently faced with difficulties due to the complexity inherent in this diversity of canal morphology. This case report describes the management of such canal variation, which requires a thorough knowledge of internal anatomy combined with detailed investigations and the operator’s skill in cleaning and shaping the canal for better prognostic outcomes.

## Introduction

One variation that is more common in mandibular second molars and has a racial predilection is the C-shaped canal arrangement. During negotiation, debridement, and obturation, the clinician may encounter difficulties due to the subtleties inherent in this variance of canal shape. Sonic and ultrasonic assistance, along with rotational and manual instrumentation to ensure each and every surface is cleaned, can effectively control this abnormal canal shape. Adjustments to the obturation procedures, such as the use of thermoplasticized obturation technique or adequate penetration of condensers for lateral condensation techniques, will ensure a three-dimensional fill of the canal system and pulp chamber-retained that pin-retained restorations such as dental amalgam or resin composites function satisfactorily as post-obturation restorations [1]. 

The C-shaped canal configuration is one example of a root canal system variant. The term "root canal" refers to the anatomical arrangement of the root and its C-shaped cross-section [2]. Cooke and Coxin (1979) published the first description of this condition in the literature [3]. According to Manning's description of the root canal anatomy of mandibular second molars, the first records of C-shaped roots and root canals date back to 1908 and 1911, respectively, and were based on an analysis of the skeletal remains of Neanderthals, the ancestors of the Mongoloid race which also includes Asian populations [3-5]. 

Melton, in 1991, proposed the following classification of C-shaped canals based on their cross-sectional shape: category I is a continuous C-shaped canal running from the pulp chamber to the apex, defines a C-shaped outline without any separation; category II is semicolon-shaped where the orifice in which dentine separates a main C-shaped canal from one mesial distinct canal; category III refers to those with two or more discrete and separate canals: subdivision I is a C-shaped orifice in the coronal third that divides into two or more discrete and separate canals that join apically; subdivision II is C-shaped orifice in the coronal third that divides into two or more discrete and separate canals in the midroot to the apex; and subdivision III is a C-shaped orifice that divides into two or more discrete and separate canals in the coronal third to the apex [6]. The prevalence of C-shaped canals varies worldwide. It has around 2.7%-7.6% prevalence in a study of the Caucasian population, in India it is around 22%, and Von Zuben et al. noted an 11% prevalence in the United States of America [7].

## Case presentation

**Figure 1:**
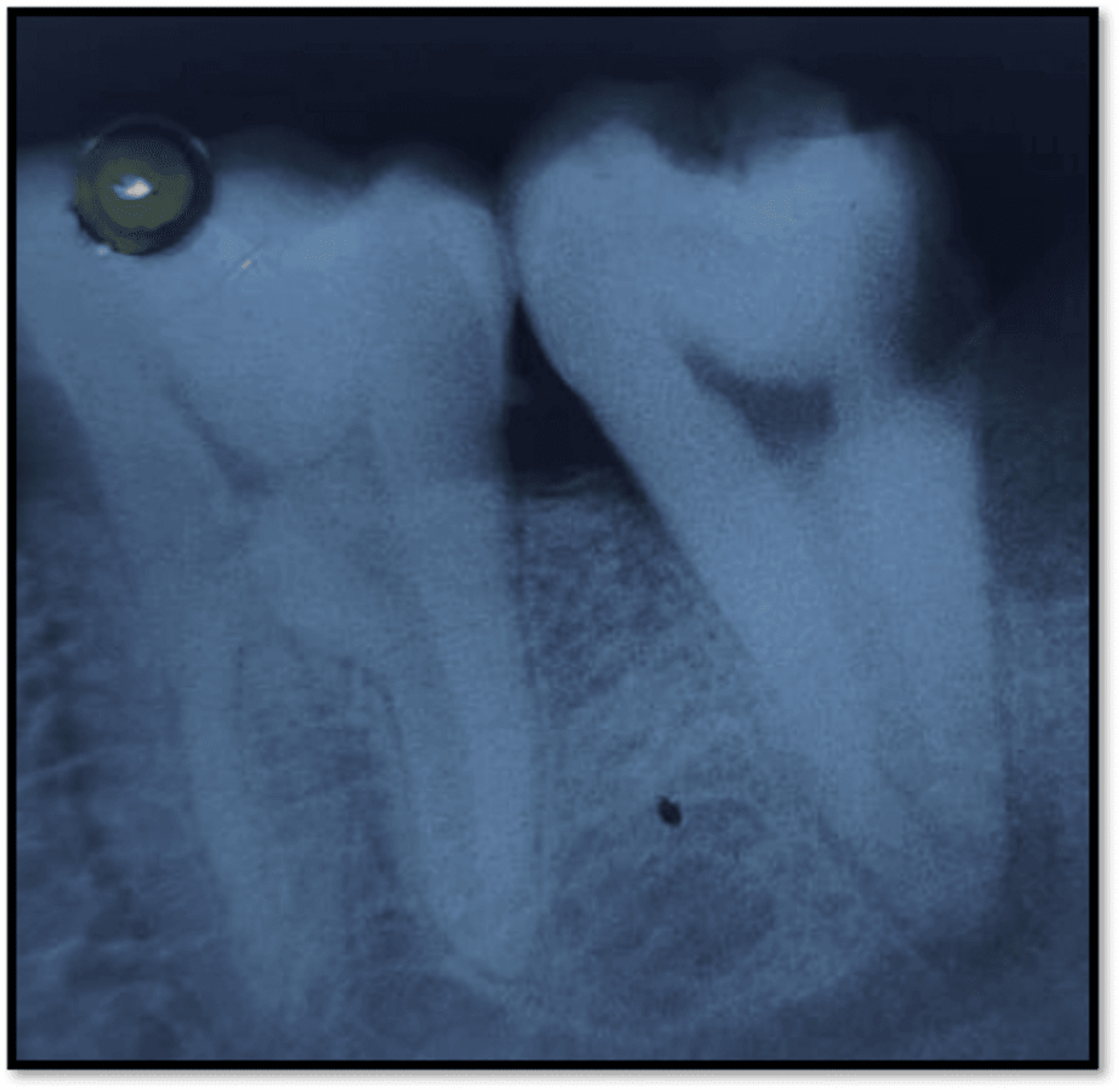
Intraoral periapical preoperative radiograph with 47 This intraoral periapical radiograph shows distoproximal caries with a second molar involving enamel, dentin, and pulp suggestive of irreversible pulpitis with 47

A 48-year-old male patient reported pain in the lower right back region of the jaw for one month. The patient was apparently alright one month back before he started experiencing pain with no relevant past medical history. The pain was spontaneous, sharp shooting, and throbbing in nature, which got aggravated during the night and was not relieved even on intake of medication. There was no history of swelling, pus discharge, or fever. Clinical examination revealed distoproximal caries with 47 which was tender on vertical percussion. Diagnostic procedures performed were neural sensibility testing and intraoral periapical radiograph. Neural sensibility testing revealed delayed response with 47. In radiographic examination, intraoral periapical radiograph of 47 revealed radiolucency involving enamel, dentin, and approaching pulp (Figure 1). Root canal treatment was planned with 47 with the expected outcome of resolution of the signs and symptoms associated with 47. 

**Figure 2:**
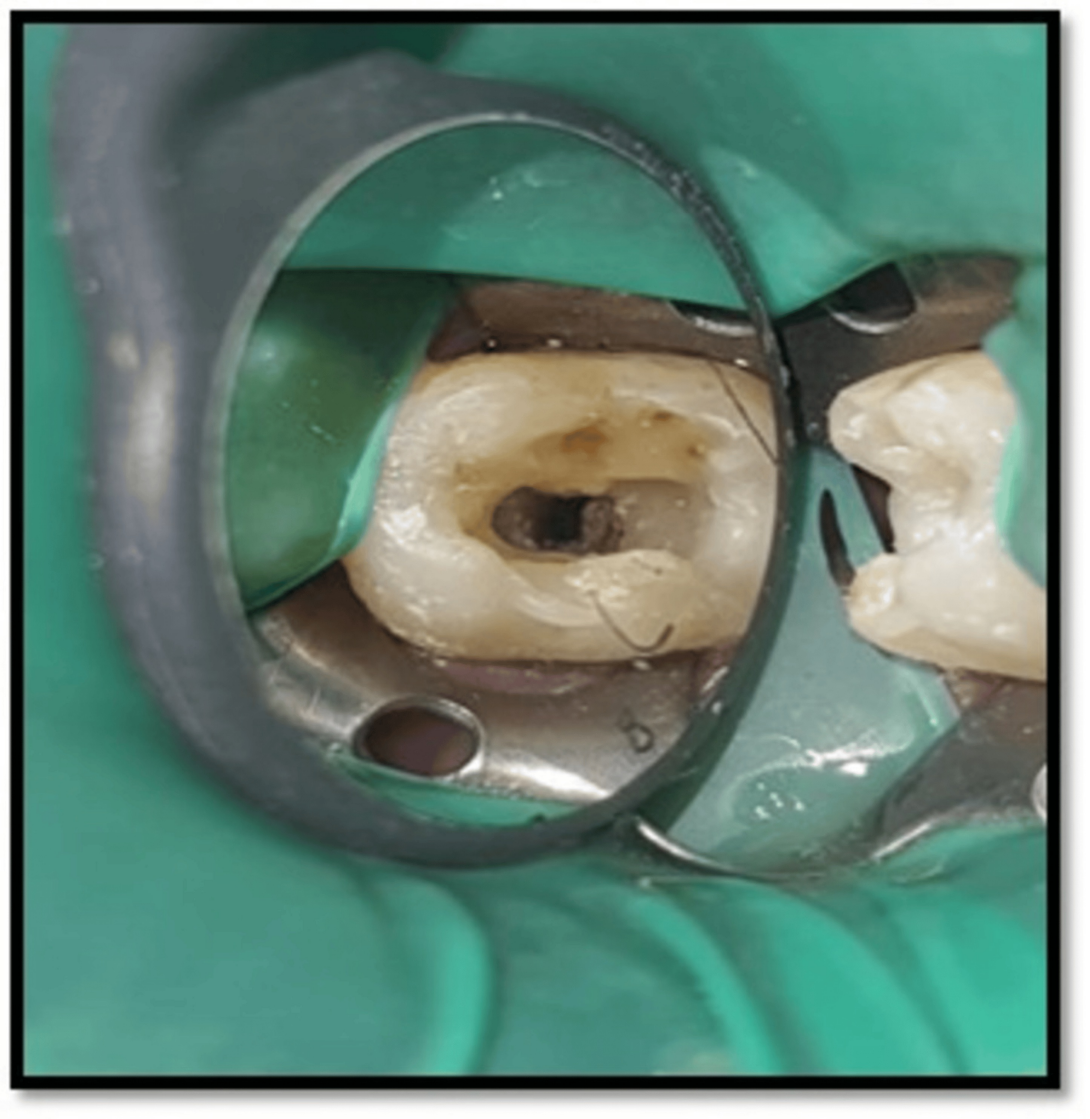
Access opening with 47 This intraoral clinical picture shows access opening with tooth number 47

Local anesthesia was achieved by administering an inferior alveolar nerve block using 2% lidocaine (1:100,000 of epinephrine). Rubber dam isolation was done with 47 caries excavation was done, followed by a pre-endodontic composite buildup with 47. Access cavity preparation was performed using BR 45 bur and modified with EX 24 bur with 47 (Figure 2).

**Figure 3:**
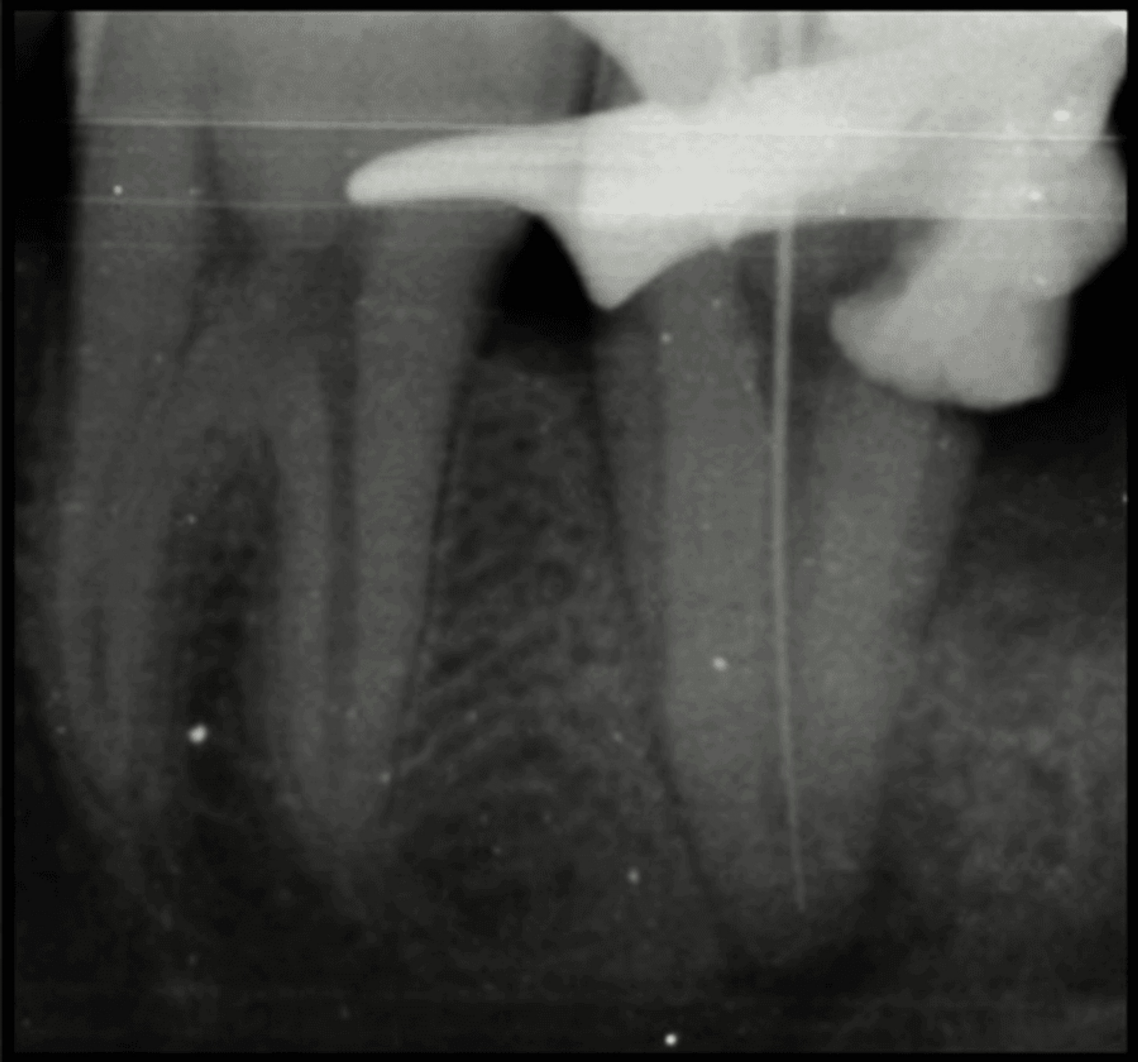
Intraoral periapical radiograph of working length determination with 47 The intraoral periapical radiograph shows a working length determination of 47 using a 15K file

Only a single oval orifice at the center of the floor of the pulp chamber was located with 47. Pulp extirpation was done. Patency was achieved using a 10K file. The canal was irrigated using saline and 5.25% Sodium hypochlorite. Working length was determined using an apex locator and was verified radiographically (21mm) (Figure 3).

**Figure 4:**
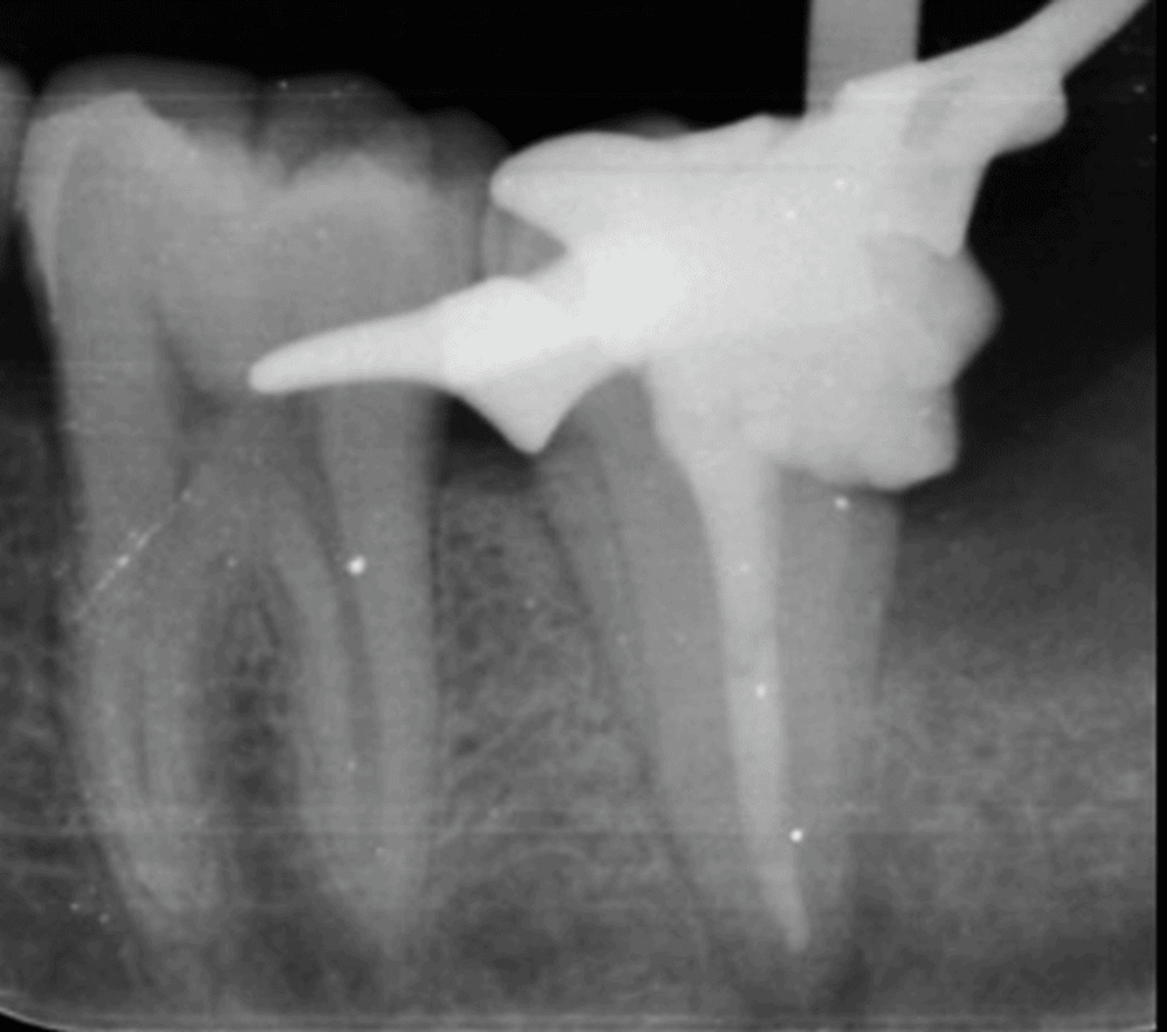
Intraoral periapical radiograph showing obturation with 47 This intraoral periapical radiograph shows obturation with 47

Biomechanical preparation was done using 2% hand files up to a 55K file, followed by step-back preparation up to 70K files. Calcium hydroxide dressing was given for seven days. After relief of symptoms, mastercone fit was assessed. Gutta-percha (GP) was placed in the canal using a warm vertical compaction technique along with nanoseal sealer, followed by backfilling of the canal using a thermoplasticized gutta-percha obturation technique. System B Cordless (Obturation II) system (KaVo Kerr, Brea, California) was used for backfilling of the canal space (Figure 4).

Post-endodontic restoration was completed using composite resin.

## Discussion

The mandibular second molar is frequently affected by a kind of taurodontism known as the C-shaped root canal arrangement, which varies anatomically in root fusion and occurrence in different groups from 2.7% to 45.5% [6]. This arrangement is a barrier to comprehensive debridement for the clinician since the root canals are connected by slits or webs with different anatomical features along the length of the root [7]. When the buccal or lingual aspects of the mesial and distal roots fuse together, a C-shaped canal is created. The two roots continue to fuse irregularly, with an interradicular ribbon keeping them joined. Two or three canals may be discovered in the C-shaped groove, or the C-shape may be continuous along the length of the root. The pulp chamber's floor is unusually anatomical in appearance and is quite deep [8].

The mandibular molars of the Indian population exhibit a broad range of complex anatomical differences [9]. Therefore, due to unusual structure and variety of shapes, molars present a challenge for root canal therapy because of the restricted access [10]. The root canal must be completely cleaned and shaped for endodontic success. Debris removal is necessary to reach this objective [11]. Due to the reduced success rate of root canal therapy, dental professionals, particularly endodontists, have been aware of the issue of teeth with C-shaped canals. It is obvious that one cannot always foresee the root canal anatomy, and this may be the cause of failure. The cornerstone of effective endodontic therapy is a thorough understanding of the anatomy of the tooth, including the quantity and duration of different root canal treatments.

Working length radiographs are more helpful than preoperative and final radiographs in identifying C-shaped canals. A true C-shaped canal is a single canal that extends from the orifice to the apex so that an instrument can pass through it without obstruction from the mesial to the distal aspect [12]. Anytime an instrument is entered into any side of the C-shaped canal in the semicolon type, it always ends in the distal foramen of the tooth, and a file implanted in this canal might explore the entire length of the C [13].

Cleaning and filling treatments are part of the process of obturation and preparation of the canal system, and these can be completed using a variety of customized approaches to manage such instances endodontically. Preparing the deep orifice and using small files with caution is necessary for the C-shaped canal. In order to prevent strip perforation, extra caution is taken when preparing the mesiobuccal, mesioligual, and distal canals. Specifically, no larger than No. 25 file should be used when preparing the isthmus. Typically, a complete debridement of the narrow canal isthmus requires small files and a generous dose of 5.25% sodium hypochlorite. Gates glidden drills should not be utilized for such tiny, connected isthmus sections; instead, cleaning should be done with a tool no bigger than size ISO 25 [14].

## Conclusions

The unusual anatomy of a mandibular second molar with a single C-shaped canal is described in this case study. Success in such aberrant situations is aided by careful analysis of the tooth under magnification, appropriate radiographic and clinical evaluation using endodontic explorers, microdebriders and the selection of a cleaning and shaping approach that is appropriate for this unique root canal anatomy. The irrigation protocol using proper activation aids such as sonic and ultrasonic activation is also crucial for the success and prognosis of C-shaped root canal anatomy.

## References

[1] Fernandes M, de Ataide I, Wagle R (2014). C-shaped root canal configuration: a review of literature. J Conserv Dent.

[2] Rahimi S, Shahi S, Lotfi M, Zand V, Abdolrahimi M, Es'haghi R (2008). Root canal configuration and the prevalence of C-shaped canals in mandibular second molars in an Iranian population. J Oral Sci.

[3] Cooke HG 3rd, Cox FL (1979). C-shaped canal configurations in mandibular molars. J Am Dent Assoc.

[4] de Paula-Silva FW, Wu MK, Leonardo MR, da Silva LA, Wesselink PR (2009). Accuracy of periapical radiography and cone-beam computed tomography scans in diagnosing apical periodontitis using histopathological findings as a gold standard. J Endod.

[5] Manning SA (1990). Root canal anatomy of mandibular second molars. Part II. C-shaped canals. Int Endod J.

[6] Melton DC, Krell KV, Fuller MW (1991). Anatomical and histological features of C-shaped canals in mandibular second molars. J Endod.

[7] von Zuben M, Martins JN, Berti L (2017). Worldwide prevalence of mandibular second molar C-shaped morphologies evaluated by cone-beam computed tomography. J Endod.

[8] Neelakantan P, Subbarao C, Subbarao CV, Ravindranath M (2010). Root and canal morphology of mandibular second molars in an Indian population. J Endod.

[9] Fan W, Fan B, Gutmann JL, Cheung GS (2007). Identification of C-shaped canal in mandibular second molars. Part I: radiographic and anatomical features revealed by intraradicular contrast medium. J Endod.

[10] Bolger WL, Schindler WG (1988). A mandibular first molar with a C-shaped root configuration. J Endod.

[11] Sarangi S, Chandak M, Sedani S, Agrawal P, Nadgouda M, Shirbhate U (2024). Unveiling the Enigma: A confluence of two case reports for the negotiation of mid-mesial canals. Cureus.

[12] Bhopatkar J, Ikhar A, Nikhade P, Chandak M, Agrawal P (2023). Navigating challenges in the management of mandibular third molars with Radix paramolaris: a case report. Cureus.

[13] Rajnekar R, Mankar N (2021). Comparative evaluation of apical debris extrusion during root canal preparation using three different rotary file systems: a study protocol. J Pharm Res Int.

[14] Singh T, Kumari M, Kochhar R, Iqbal S (2022). Prevalence of C-shaped canal and related variations in maxillary and mandibular second molars in the Indian Subpopulation: a cone-beam computed tomography analysis. J Conserv Dent.

